# Synthesis and Meta-analysis of 3 Randomized Trials Conducted in Burkina Faso, Ghana, and Uganda Comparing the Effects of Point-of-Care Tests and Diagnostic Algorithms Versus Routine Care on Antibiotic Prescriptions and Clinical Outcomes in Ambulatory Patients <18 Years of Age With Acute Febrile Illness

**DOI:** 10.1093/cid/ciad324

**Published:** 2023-07-25

**Authors:** Piero Olliaro, Juvenal Nkeramahame, Philip Horgan, Halidou Tinto, François Kiemde, Rita Baiden, Alexander Adjei, James Kapisi, Heidi Hopkins, Olawale Salami, Catrin E Moore, Sabine Dittrich, Stephan Weber, Stefano Ongarello, the ADIP study group, Phyllis Awor, Deborah Ekusai-Sebatta, Heidi Hopkins, David Kaawa-Mafigiri, James Kapisi, Freddy Eric Kitutu, Elizeus Rutebemberwa, Asadu Sserwanga, Alexander Adjei, Rita Baiden, Vida Kukula, Adélaïde Compaoré, François Kiemde, Halidou Tinto, Daniel Valia

**Affiliations:** Nuffield Department of Medicine, University of Oxford, Oxford, UK; FIND, Geneva, Switzerland; FIND, Geneva, Switzerland; FIND, Geneva, Switzerland; Nuffield Department of Medicine, Big Data Institute, University of Oxford, Oxford, UK; Evidence & Impact Oxford, Oxford, UK; Clinical Research Unit of Nanoro, Institut de Recherche en Sciences de La Santé, Nanoro, Burkina Faso; Clinical Research Unit of Nanoro, Institut de Recherche en Sciences de La Santé, Nanoro, Burkina Faso; INDEPTH-Network, Accra, Ghana; Dodowa Health Research Centre, Dodowa, Ghana; Dodowa Health Research Centre, Dodowa, Ghana; Infectious Diseases Research Collaboration, Kampala, Uganda; London School of Hygiene & Tropical Medicine, London, UK; FIND, Geneva, Switzerland; Nuffield Department of Medicine, Big Data Institute, University of Oxford, Oxford, UK; Centre for Neonatal and Paediatric Infection, Institute for Infection and Immunity, St George’s University of London, London, UK; FIND, Geneva, Switzerland; Centre for Tropical Medicine and Global Health, Nuffield Department of Medicine, University of Oxford, Oxford, UK; Deggendorf Institute of Technology, European Campus Rottal Inn, Pfarrkirchen, Germany; FIND, Geneva, Switzerland; FIND, Geneva, Switzerland

**Keywords:** antibiotic prescriptions, point-of-care diagnostic tests, febrile illnesses, diagnostic algorithms

## Abstract

This meta-analysis included 3 randomized trials conducted in sub-Saharan Africa comparing the effects of point-of-care tests and diagnostic algorithms versus routine care on antibiotic prescriptions and clinical outcomes in ambulatory patients presenting at outpatient facilities with acute uncomplicated febrile illness.

This article summarizes the characteristics and outcomes of 3 randomized trials (conducted in Burkina Faso, Ghana, and Uganda) comparing the effects of point-of-care tests and diagnostic algorithms with those of routine care on antibiotic prescriptions and clinical outcomes in ambulatory patients presenting at outpatient facilities with acute uncomplicated febrile illness. These trials are part of the Antimicrobial Resistance (AMR) Diagnostic Use Accelerator Program (ADIP), set up by FIND to identify practical ways to help prevent further development and spread of antimicrobial resistance and improve the quality of care for patients with acute febrile illnesses in low-resource settings. The background, objectives, and methods of this program are presented in the introductory article in this issue; individual country results are presented separately in the relevant articles in this supplement (see Kiemde et al, Adjei et al, and Kapisi et al [1–3]).

## METHODS

Details of clinical trial methods and data management are published in the protocol [5], as well as in the introductory article and the individual study reports in this issue (see Olliaro et al, Kiemde et al, Adjei et al, and Kapisi et al [1–4]). This meta-analysis is restricted to children and young people <18 years of age. (Adults were also recruited in Uganda, but they are excluded from this meta-analysis for reasons of consistency across study sites; the complete study results are provided by Kapisi et al. Descriptive statistics are used for baseline and demographic characteristics by country and overall. Categorical values are summarized as percentages, continuous variables as means with standard deviation and or medians with interquartile range.

In this article, we present individual study data and pooled estimates of relative (risk ratio [RR]) and absolute (risk difference [RD]) effects with 95% confidence intervals (CIs). The same overall estimates were obtained both by individual participant data and aggregated data meta-analysis with a fixed effect on the country. RevMan 5.4.1 software was used to generate aggregated estimates and forest plots [6].

In addition, a multivariable log-link model, has been performed, modeling the antibiotic prescription status accounting for site-specific prescription frequencies per group, adjusted for basic demographic properties and diagnostic information available for both arms (ie, malaria rapid diagnostic test [RDT] results and respiratory or nonrespiratory diseases diagnosed).

## RESULTS

A total of 4715 ambulatory patients <18 years of age, presenting with fever for <7 days, were recruited at 7 sites in 3 African countries. The study population was well balanced in terms of country contribution (1718 patients [36.4%] in Burkina Faso, 1512 [32.1%] in Ghana, and 1485 [31.5%] in Uganda), randomization (2320 [49.2%] randomized to the intervention arm and 2395 [50.8%] to routine care), and sex. The overall median age was 3 years (Table 1).

**Table 1.** Patient Demographics and Baseline Characteristics

Characteristic	Patients by Treatment Group, No. (%)^a^											
	Burkina Faso			Ghana			Uganda			All 3 Countries		
	Total	Intervention	Control	Total	Intervention	Control	Total	Intervention	Control	Total	Intervention	Control
Total enrolled	1718 (36.4)	856 (49.8)	862 (50.2)	1512 (32.19)	761 (50.3)	751 (48.7)	1485 (31.5)	703 (47.3)	782 (52.7)	4715	2320 (49.2)	2395 (50.8)
Sex ratio, % male:% female	48.9:51.1	48.5:51.5	49.3:50.7	46.5:53.5	47.6:52.4	45.4:54.6	47.1:52.9	47.5:52.5	46.8:53.2	49.4:50.6	49.4:50.6	49.4:50.6
Age, median (IQR), y	2.8 (1.5–4.7)	2.8 (1.5–4.6)	2.8 (1.4–4.8)	2 (1–4)	2 (1–4)	2 (1–4)	5 (2.1­–8)	4 (2.4–8)	5 (2–8)	3 (1.5–6)	3 (1.5–6)	3 (1.5–6)
Age group, y												
<5	1302 (75.8)	653 (76.3)	649 (75.3)	1154 (76.3)	584 (76.7)	570 (76.0)	741 (30.9)	364/1189 (30.6)	377/1211 (31.1)	3197 (56.8)	1601/2806 (57.1)	1596/2824 (56.5)
5 to <10	305 (17.8)	146 (17.0)	159 (18.4)	243 (16.1)	118 (15.5)	125 (16.6)	432 (18.0)	196/1189 (16.5)	236/1211 (19.5)	980 (17.4)	460/2806 (16.4)	520/2824 (18.4)
10 to <18	111 (6.5)	57 (6.7)	54 (6.3)	115 (7.6)	59 (7.8)	56 (7.5)	312 (13.0)	143/1189 (12.0)	169/1211 (14.0)	538 (9.6)	259/2806 (9.2)	279/2824 (9.9)
Respiratory diagnosis	846 (49.2)	470 (54.9)	376 (43.6)	1110 (73.4)	584 (76.7)	526 (70.0)	750/1410 (53.29)	468/723 (66.6)	282/707 (39.9)	2706/4640 (58.3)	1522 (65.6)	1184/2320 (51)
Malaria RDT positive	1285 (74.8)	646 (75.5)	639 (74.1)	162/1434 (11.3)	84/738 (11.4)	78/696 (11.2)	740/1341 (55.2)	375 (53.3)	365/638 (57.2)	2187/4493 (48.7)	1105/2297 (48.1)	1082/2196 (49.3)

Abbreviations: IQR, interquartile range; RDT, rapid diagnostic test.

Data represent no. (%) of patients unless otherwise specified. Denominators are provided where they differ from the column’s “total enrolled” number.

### Baseline Characteristics

Overall, 2076 of 4640 patients (44.7%) of patients had a respiratory disease diagnosed (either upper or lower respiratory tract), but with significant variations across countries; these patients included 846 of 1718 (49.2%) in Burkina Faso, 750 of 1410 (53.3%) in Uganda, and 1110 of 1512 (73.4%) in Ghana. A respiratory condition was also diagnosed significantly more frequently in the intervention group: on aggregate in 1522 of 2320 (65.6%) versus 1184 of 2320 (51%) in the control arm (*P* < .001). At the country level, the difference was statistically significant in Burkina Faso (*P* = .006) and Uganda (*P* < .001) but not Ghana (*P* = .2).

In these malaria-endemic areas, malaria testing was done systematically in 4493 of 4715 patients (95.3%) and done more often, by a small but significant margin, in the intervention group: in 2297 of 2320 patients (99%) in the intervention and 2196 of 2395 (97%) in the control group (*P* < .001). The overall malaria-positive RDT proportion was 49.7%, ranging from 11.2% in Ghana to 55.2% in Uganda and 74.8% in Burkina Faso. RDT positivity rates were similar in the 2 arms: 1105 of 2297 (48.1%) in the intervention versus 1082 of 2196 (49.3%) in the control groups overall, and also within each country. Rates varied over time with the transmission season (Supplementary Table 1).

### Outcomes

#### Clinical Outcome

**Figure 1.**
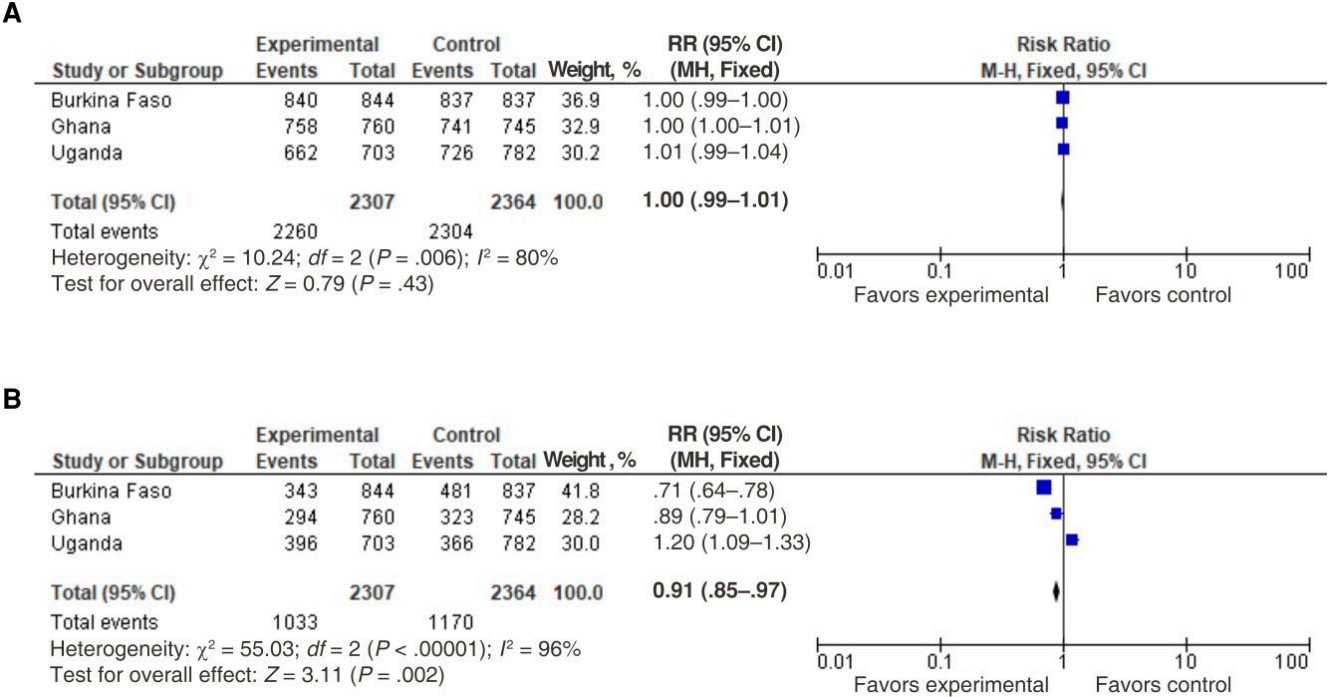
Clinical outcome (*A*) and antibiotic prescription rates (*B*). Note that no follow-up information was available for 37 patients in Burkina Faso (12 in the intervention and 25 in the control group 25) and 7 in Ghana (1 in the intervention and 6 in the control group). Abbreviations: CI, confidence interval; MH fixed, Mantel-Haenszel fixed-effect method; RR, risk ratio.

More than 97% of patients experienced a favorable clinical outcome at the day 7 follow-up visit, defined as normal body temperature and reporting improvement or resolution of day 0 symptoms, with no difference between arms in terms of both relative (Figure 1*A*) and absolute effects (Supplementary Figure 1*A*) both at the country level and for aggregated data. The proportion of patients experiencing favorable clinical outcomes was similar between female patients (97.7% in the intervention and 97.4% in the control group) and male patients (98.2 and 97.5%, respectively). Clinical outcomes also did not differ between patients who received antibiotics and those who did not.

#### Antibiotic Prescriptions

The relative and absolute effects on antibiotic prescriptions are summarized in Table 2, Figure 1*B*, and Supplementary Figure 1*B*. The pooled estimated RR (95% CI) was 0.91 (.85–.97) and the pooled estimated RD was −0.05 (−0.02 to −0.07), translating into a number needed to test (NNT) of 20 (14–50) (defined as the number needed to test to prevent 1 antibiotic prescription [or to lead to 1 additional antibiotic prescription if positive]).

**Table 2.** Summary Outcomes: Antibiotic Prescriptions by Country and Overall

Country	Patients With ATBs Prescribed/Total in Group, No. (%)		Effect		
	Intervention Group	Control Group	Relative: Risk Ratio (RR) (95% CI)	Absolute: Risk Difference (RD) (95% CI)	No. Needed to Test^a^
Burkina Faso	343/844 (40.6)	481/837 (57.5)	29% Reduction (−22% to −36%)	17 Fewer ATB Rx per 100 patients tested (12–22 fewer)	1 Fewer ATB Rx for every 6 patients tested (range: 8 to 5 patients)
Ghana	294/760 (38.7)	323/745 (43.4)	11% Reduction (−21% to 1%)	5 Fewer ATB Rx per 100 patients tested (0–10 fewer)	1 Fewer ATB Rx for every 21 patients tested (range: from 1 fewer for every 10 patients, to 1 more for every 340 patients)
Uganda	396/703 (56.3)	366/782 (46.8)	20% Increase (9%–33%)	10 More ATB Rx per 100 patients tested (4–15 more)	1 More ATB Rx for every 10 patients tested (range: 25 to 7 patients)
All countries	1033/2307 (44.8)	1170/2364 (49.5)	9% Reduction (−3% to −15%)	5 Fewer ATB Rx per 100 patients tested (2–7 fewer)	1 Fewer ATB Rx for every 20 patients tested (range: 50 to 14 patients)

Abbreviations: ATB, antibiotic; CI, confidence interval; RD, risk difference; RR, risk ratio; Rx, prescription(s).

Number needed to test to prevent 1 more ATB Rx if results were negative (or lead to 1 more if they were positive).

#### Antibiotic Prescriptions in Subgroups

##### Antibiotic Prescriptions by Age and Sex

Effects on antibiotic prescriptions varied with participant age but not by sex. There was a significant decrease in antibiotic prescriptions overall among children <5 years old (pooled estimated RR, 0.84 [95% CI, .78–.90]), ranging from a significant decrease in Burkina Faso (0.67 [.61–.75]) and Ghana (0.86 [.75–.98]) to a significant increase in Uganda (1.16 [1.02–1.33]). In the age group from 5 to <10 years, there was a significant increase overall (pooled RR, 1.23 [95% CI, 1.06–1.42]), which was significant at the country level, however, only for Uganda. Finally, there was no significant difference in the age group from 10 to <18 years (RR, 0.91 [95% CI, .76–1.09]) (Supplementary Figure 2). Effects did not differ between female and male patients; among female patients, overall antibiotic prescription rates were 43.9% versus 49.1% in the intervention and control groups, respectively, compared with 45.7% versus 49.9% in male patients.

##### Antibiotic Prescriptions by Clinical Presentation—Respiratory Disease

There was a significant reduction in antibiotic prescriptions among patients with a diagnosis of respiratory disease in the intervention arm, with a pooled estimated RR of 0.77 (95% CI, .72–.82); at the country level this reduction was statistically significant in Burkina Faso (0.58 [.53–.64]) and Ghana (0.84 [.73–.96]) but not in Uganda (1.03 [.91–1.18]). There was no difference between arms among those with nonrespiratory presentation (RR, 1.05 ]95% CI, .93–1.18]) and this was reflected across all countries. (Table 3 and Supplementary Figure 3).

**Table 3.** Summary Outcomes: Antibiotic Prescriptions by Malaria and Respiratory Disease Status

Malaria RDT and Disease Status	Patients With ATBs Prescribed/Total in Group, No. (%)		Effect		
	Intervention Group	Control Group	Relative: RR (95% CI)	Absolute: RD (95% CI)	No. Needed to Test^a^
Malaria RDT negative	504/1187 (42.5)	669/1107 (60.4)	30% Reduction (−24% to −35%)	18 Fewer ATB Rx per 100 patients tested (14–22 fewer)	1 Fewer ATB Rx for every 6 patients tested (range: 7 to 5 patients)
Respiratory disease	747/1511 (49.4)	743/1177 (63.1)	23% Reduction (−18% to −28%)	15 Fewer ATB Rx per 100 patients tested (11–18 fewer)	1 Fewer ATB Rx every 7 patients tested (range: 9 to 6 patients)
Malaria RDT negative and respiratory disease	390/938 (41.6)	443/751 (59)	31% Reduction (−24% to −33%)	18 Fewer ATB Rx per 100 patients tested (13–22 fewer)	1 Fewer ATB Rx for every 6 patients tested (range: 8 to 5 patients)

Abbreviations: ATB, antibiotic; CI, confidence interval; RD, risk difference; RDT, rapid diagnostic test; RR, risk ratio; Rx, prescription(s).

Number needed to test to prevent 1 more ATB Rx if results were negative.

##### Antibiotic Prescriptions by Malaria Diagnosis

The intervention arm had significantly fewer antibiotic prescriptions among those with a negative malaria test (RR, 0.70 [95% CI, .65–.76]), and this was true in all 3 countries (RR, 0.46 in Burkina Faso, 0.69 in Uganda, and 0.85 in Ghana). Instead, antibiotic prescription among patients with diagnosed malaria were higher in the intervention than in the control groups: the pooled estimated RR was 1.18 (95% CI, 1.07–1.29) but ranged from a decrease (0.86 [.76–.98] in Burkina Faso) to an increase (1.92 [1.64–2.26] in Uganda) (Table 3 and Supplementary Figure 4).

##### Antibiotic Prescriptions by Respiratory Disease and Malaria Diagnosis

When analyzing antibiotic prescriptions by combined malaria and respiratory diagnoses, the greatest benefit was for patients with respiratory disease who are malaria negative (pooled RR [95% CI], 0.70 [.63–.76]; Burkina Faso, 0.43 [.35–.52]; Ghana, 0.81 [.71–.94]; and Uganda 0.76 [.63–.91]). A pooled summary estimate in favor of the intervention was also found for patients with respiratory disease who are malaria positive (RR, 0.81 [95% CI, .74–.89]), and those with unknown status (0.86 [.77–.95]). By contrast, patients with malaria and nonrespiratory disease had an overall increase in antibiotic prescriptions (RR, 1.52 [95% CI, 1.27–1.83]). These effects are similar across the different age categories. (see Table 3 and Supplementary Figure 5 for all ages and Supplementary Figure 6 for children <5 years old). Effects also varied over time and within the same country (Supplementary Table 2).

##### Multivariable Analysis

The multivariable analysis showed consistent results with the univariable analyses presented above, with an overall estimated RR of 0.87 (95% CI, .81–.93; *P* < .001).

#### Use and Results of Tests in the Intervention Group

In the intervention arm, a malaria RDT was performed in 99% of patients overall, white blood cell (WBC) total and neutrophil counts in 96.9%, and C-reactive protein (CRP) in 96.6%; these values were consistent across the 3 countries (Supplementary Table 3). Other tests were applied far less, and less consistently, with huge variation between countries (Burkina Faso was the most conservative and Ghana the most systematic): influenza (69.6% of patients), typhoid (61.2%), respiratory syncytial virus (RSV), group A *Streptococcus* (GAS), and *Streptococcus pneumoniae* (44.4%, 41.4%, and 32.8% respectively), and WBC esterase and nitrate (approximately 21% each) (Supplementary Table 3).

Test results also varied widely across the 3 countries in the intervention arm. For instance, malaria incidence rates were very high in Burkina Faso (75.5%) compared with Uganda (53.3%) and Ghana (11.4%), a difference that was mirrored by CRP levels (median, 28.6, 17.3, and 1.7, respectively); the overall median CRP level was 11.5 µg/mL (interquartile range, 1.0–49.4 µg/mL), with values ranging from 1 to 150 µg/mL (Supplementary Table 3).

CRP levels differed between malaria-positive and malaria-negative patients (malaria is expected to be associated with elevated CRP levels) (Supplementary Figure 7). Overall, approximately 33% of cases fell in each of 3 preestablished CRP categories (<20, 20–80 and >80 mg/L) among malaria RDT–positive patients, compared with 82%, 12% and 6%, respectively, among malaria RDT–negative patients. CRP levels <10 mg/L were recorded in 70% of patients.

WBC total counts were similar in the 3 countries. It is not possible to derive the true incidence of infections like typhoid, streptococcus, influenza, and RSV, as the tests were applied so variably, but the proportions of positive test results were generally low. In Ghana, with the tests done systematically, independent of clinical presentation, influenza was detected in 10% of cases, GAS in 4.6%, RSV in 2.8%, and typhoid in 1.6% (Supplementary Table 3).

Antibiotics were generally prescribed after positive results of blood test for typhoid, throat swab test for GAS, or urine test for *S. pneumoniae*, with few exceptions, but less so for WBC esterase and nitrate tests. Overall, antibiotics were prescribed in 45% and 53% of influenza-positive and RSV-positive results, respectively (Supplementary Table 4). Antibiotic prescriptions in the intervention arm increased with WBC counts and CRP and neutrophil levels, but rates differed across countries (Supplementary Table 5).

## DISCUSSION

Taken together, the results of these studies indicate that antibiotic prescriptions can be reduced without worsening clinical outcomes in children and young people with acute uncomplicated fever seen at outpatient facilities in sub-Saharan Africa. The effectiveness of the intervention in reducing unnecessary antibiotic prescriptions is highest in patients with nonmalarial fever and respiratory symptoms and in children <5 years of age.

On aggregate, the intervention resulted in a 9% reduction in antibiotic prescriptions (from as little as 3% to as much as 15%) relative to routine procedures and led to 1 fewer antibiotic prescription in every 20 patients (from as many as 50 to as few as 14). There was substantial heterogeneity in the size and direction of effects (eg, for example, a 29% reduction for Burkina Faso, with 1 fewer antibiotic prescription for every 6 patients tested, vs a 20% increase for Uganda, with 1 more prescription for every 10 tested), which means that these results may not be universally applicable as such. Despite this heterogeneity—mostly on account of differences in patients’ demographics and presenting conditions (eg, rates of malaria and respiratory disease) and physicians’ approaches (use and interpretation of tests, clinical judgment, and case management decisions)—prescribing effects in patients with fever not caused by malaria and/or with respiratory manifestations are consistently seen in the 3 countries, though with varying effect sizes. These results were confirmed in a multivariate meta-analysis that showed an overall relative reduction of 13% (range, 7%–19%).

The intervention led to 14–22 fewer prescriptions per 100 patients tested (ie, 1 fewer prescription for every 5–7 patients tested) in patients with negative malaria RDT results; 11–18 fewer prescriptions per 100 (1 fewer for every 6–9 patients tested) in those with respiratory disease; and 13–22 fewer prescriptions per 100 (1 fewer for every 8–5 patients tested) in those who had both respiratory disease and a negative malaria test result. Of note, these effects were similar across the different age categories.

The marked effects on antibiotic prescriptions in patients without malaria and those with respiratory disease are particularly important, because both of these conditions represent a substantial proportion of patients seeking care at ambulatory health facilities—respiratory disease in more than two-thirds, nonmalarial fever in about half, and both conditions occurring together in nearly half. These findings are particularly important as the widespread use of malaria RDTs and application of the World Health Organization's test-and-treat malaria guidelines [7] can paradoxically increase antibiotic prescriptions in nonmalaria cases, in the absence of valid alternatives [8,9].

Operationally, these results offer a simple system for triaging patients using a malaria RDT—which is already routinely done—and clinical examination, to help identify the groups to target and those in which the intervention may not result in the intended effects (ie, for patients with nonrespiratory disease or with a positive malaria result), unless the use of other tests is optimized. The data generated from these studies will prove useful for our understanding of how to tailor diagnostic procedures to the local situation in order to reduce unnecessary antibiotic use and provide better care in a sustainable way, in conjunction with more accurate surveillance of the prevailing causes of infections in a given area to identify the necessary tests to apply.

The study was not intended to provide “a one-size-fit-all package” to be universally applied but rather to test the effects, use, and utility of a selection of available point-of-care diagnostics within the framework of a clinical decision-making algorithm, which healthcare providers were free to apply at their discretion. For this reason, it has proved challenging to identify how the available tests were used in the decision-making process by the different operators in varied epidemiological conditions.

Among the battery of tests provided, malaria test, CRP levels, disease and WBC total and differential counts were applied systematically in the intervention groups in all 3 countries, allowing comparisons across countries. Burkina Faso had the highest incident rates of malaria and, logically, the highest CRP levels; Ghana had the lowest of both. By contrast, we see different approaches to using the other tests, with Burkina Faso being the most conservative, Ghana using several tests systematically, and Uganda in between. Therefore, the proportions testing positive can hardly be compared across the 3 countries.

As expected, CRP levels were higher in malaria-positive than in malaria-negative patients—which makes CRP unsuited for distinguishing between bacterial and nonbacterial concomitant infections in the presence of malaria. Unexpectedly, however, one-third of malaria RDT–positive cases had CRP levels <20 mg/L.

In order to optimize this intervention, critical questions must be addressed regarding the suitability of available tests and how their use would translate into changes in antibiotic prescription practices. This study confirms that CRP values can contribute to rationalizing antibiotic prescriptions and that WBC total and differential counts are of limited added value.

Of the several host biomarkers investigated to help distinguish between bacterial and nonbacterial causes of acute febrile illnesses [10], CRP is the most widely used and studied [11]. A systematic review and meta-analysis of various biomarkers concluded that CRP has good accuracy in distinguishing between bacterial and nonbacterial infections (summary area under the receiver operating characteristic curve, 0.77 [CI .73–.81]), but the optimal cutoff could not be assessed [12]. A subsequent study in nonmalarial febrile inpatients in Italy found that CRP levels with a cutoff of 11 mg/L had the best accuracy in distinguishing between bacterial and nonbacterial infections (area under the receiver operating characteristic curve, 0.90), followed by fibrinogen (0.85) and WBC total counts and neutrophil counts (0.80). No significant improvement was found when combining biomarkers [13].

When used for acute respiratory infections in primary care settings (mostly in high-income countries), the use of CRP testing produced a reduction in antibiotic prescriptions over routine care at the time of consultation (RR, 0.79 [95% CI, .7–.9]; number needed to test to benefit, 8) but also generating significantly more reconsultations within 30 days (number needed to test to harm, 27) [14]; in our study we did not follow up patients up beyond day 7. Another systematic review showed consistent results in ambulatory care services (RR, 0.81 [95% CI, .71–.92]), with improvements in effects when guidance on antibiotic prescribing was provided (0.68 [.63–.74] in adults and 0.56 [.33–.95] in children) [15].

These results are consistent with a previous Cochrane systematic review that found an RR of 0.78 (95% CI, .66–.92) for antibiotic prescriptions with CRP testing versus routine care overall but no significant effect when restricting the analysis to randomized trials (RR 0.90, 95% CI .80–1.02) [16]. In a context closer to our study conditions, ie, in children <5 years of age with acute febrile illness in an outpatient clinic in Tanzania, CRP testing in combination with procalcitonin and other tests (as directed by an electronic algorithm) produced a 43% reduction in clinical failure and a reduction in antibiotic use (RR, .39 [95% CI, .33–.45]) compared with the use of an electronic algorithm derived from the Integrated Management of Childhood Illness guidelines [17]. However, it should be noted that antibiotic prescription rates in this study were much lower than in our settings (11.5% and 29.5%, respectively, in the 2 arms) [17]. Yet even in the groups where significant gains were obtained, antibiotic prescriptions in the 3 studies remain considerably higher than expected based on CRP levels—8 patients in 10 overall had CRP levels <20 mg/L, and 7 in 10 had levels <10 mg/L, which would normally indicate a nonbacterial infection, thus not requiring antibiotics.

This study is otherwise less informative as to the role of the other tests provided and their use by the teams. While a positive test indicating a bacterial infection was almost systematically followed by antibiotic treatment, a considerable proportion of patients with negative tests and low CRP levels and WBC counts were still prescribed antibiotics on clinical presumption. For instance, patients with CRP levels <20 mg/L should not have been prescribed antibiotics unless they were <5 years old and met World Health Organization criteria for pneumonia, according to the algorithm. However, 38% of these patients did receive an antibiotic, though only a minority of them had also been found positive for typhoid (3.4%), GAS (5.1%), or *S. pneumoniae* (9.1%), possibly justifying less than half of the antibiotic prescriptions in this category.

In conclusion, these studies demonstrate that, if correctly applied, interventions combining clinical decision-making algorithms and point-of-care tests can rationalize antibiotic prescriptions without compromising clinical outcomes. There is a clear signal that this approach is particularly adapted to patient groups who represent a substantial proportion of ambulatory patients, like those with a nonmalarial fever, respiratory disease, or both and generally in children <5 years old. Triaging patients based on clinical presentation (respiratory or not) and results of a malaria RDT is relatively simple and inexpensive and is part of routine care, even in peripheral health centers. More extensive investigations will be required to refine the intervention in terms of both test selection and the use of tests by healthcare workers to decide how to treat children presenting at outpatient clinics with an acute febrile illness. A deeper understanding of prescribers’ behaviors and decision-making processes is also necessary in order to adapt training and promote buy-in.

## Supplementary Data

Supplementary materials are available at *Clinical Infectious Diseases* online. Consisting of data provided by the authors to benefit the reader, the posted materials are not copyedited and are the sole responsibility of the authors, so questions or comments should be addressed to the corresponding author.

## Supplementary Material

ciad324_Supplementary_Data

## Notes

***ADIP study group.*** Uganda: Phyllis Awor, Deborah Ekusai-Sebatta, H. H., David Kaawa-Mafigiri, J. K., Freddy Eric Kitutu, Elizeus Rutebemberwa, and Asadu Sserwanga. Ghana: A. A., Emmanuel Arthur, Elizabeth Awini, R. B., Vida Kukula; Clement Tetteh Narh, Gabriel Odonkor, Selase Odopey, and John Williams. Burkina Faso: Adélaïde Compaoré, F. K., H. T., and Daniel Valia.

***Author contributions.*** All authors were involved in the drafting and revising of the manuscript and approved the final version for submission.

***Financial support*.** This work was supported by the Swiss Agency for Development and Cooperation, the Federal Ministry of Economic Cooperation and Development, and the Department for International Development, United Kingdom, now the Foreign, Commonwealth and Development Office, United Kingdom.

***Supplement sponsorship.*** This article appears as part of the supplement “Using Diagnostic Tools to Support Antimicrobial Stewardship and Improve Outcome in Resource-Limited Contexts,” sponsored by FIND.
