# Supplementary material for: Synthesis and Meta-analysis of 3 Randomized Trials Conducted in Burkina Faso, Ghana, and Uganda Comparing the Effects of Point-of-Care Tests and Diagnostic Algorithms Versus Routine Care on Antibiotic Prescriptions and Clinical Outcomes in Ambulatory Patients <18 Years of Age With Acute Febrile Illness
Source: Clin Infect Dis. 2023 Jul 25;77(Suppl 2):S199–205. doi: 10.1093/cid/ciad324 (PMC10368413; doi:10.1093/cid/ciad324)
Supplement: ciad324_Supplementary_Data [file ciad324_supplementary_data.docx]

# APPENDICES

## Supplementary figures

SF1. Clinical outcome (A) and antibiotic prescriptions (B) - Risk difference

A


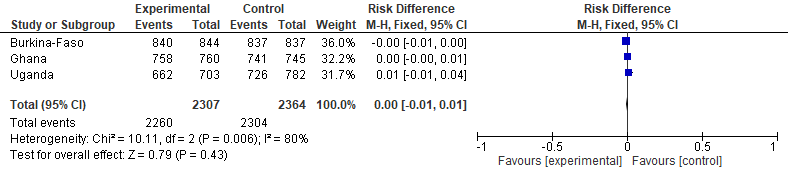
 B


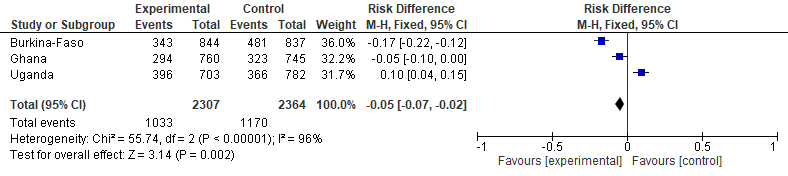


CI, confidence interval; M-H, Mantel-Haenszel method.

SF2. Antibiotic prescriptions by age-group – relative (A) and absolute (B) effects

A


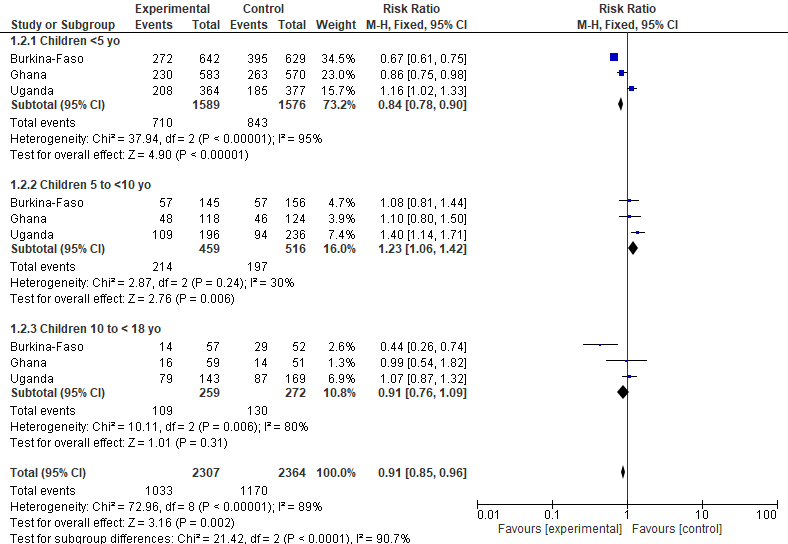


CI, confidence interval; M-H, Mantel-Haenszel method; yo, years old.

B


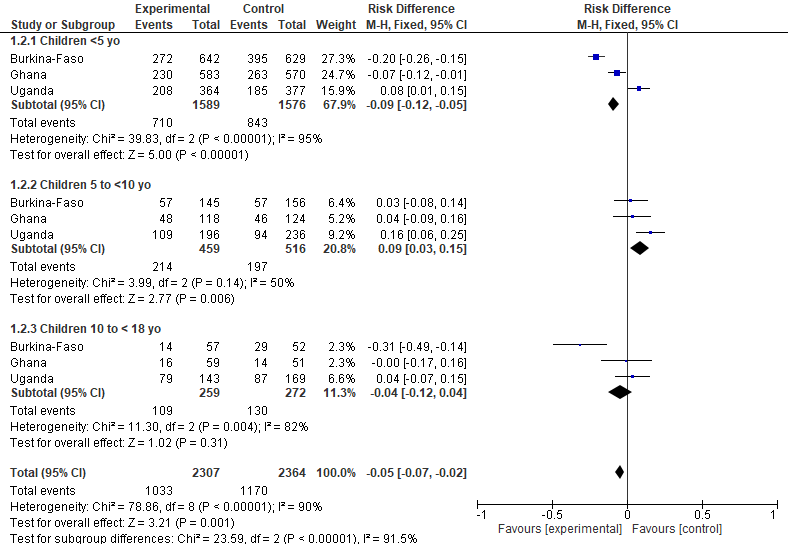


CI, confidence interval; M-H, Mantel-Haenszel method; yo, years old.

SF3. Antibiotic prescriptions by respiratory disease – relative (A) and absolute (B) effects

A


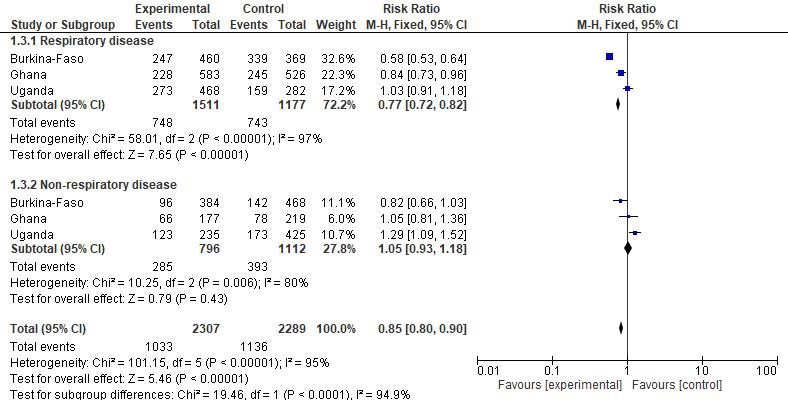


CI, confidence interval; M-H, Mantel-Haenszel method.

B


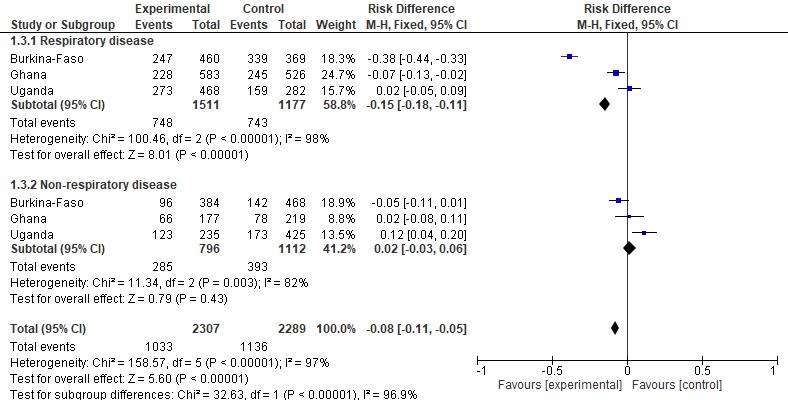


CI, confidence interval; M-H, Mantel-Haenszel method.

SF 4 Antibiotic prescriptions malaria RDT result – relative (A) and absolute (B) effects

A


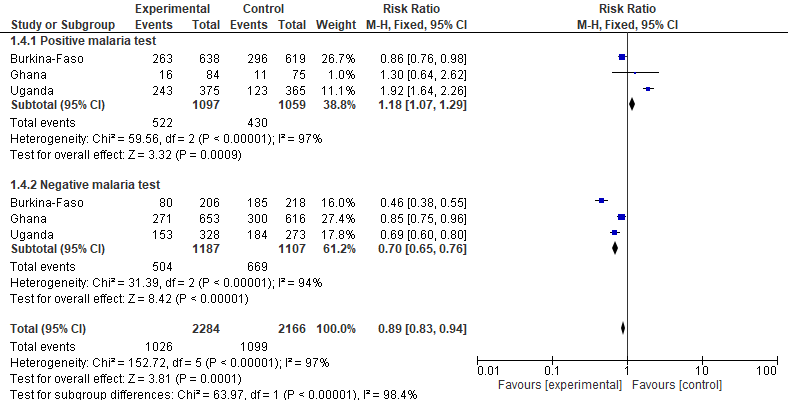


CI, confidence interval; M-H, Mantel-Haenszel method.

B


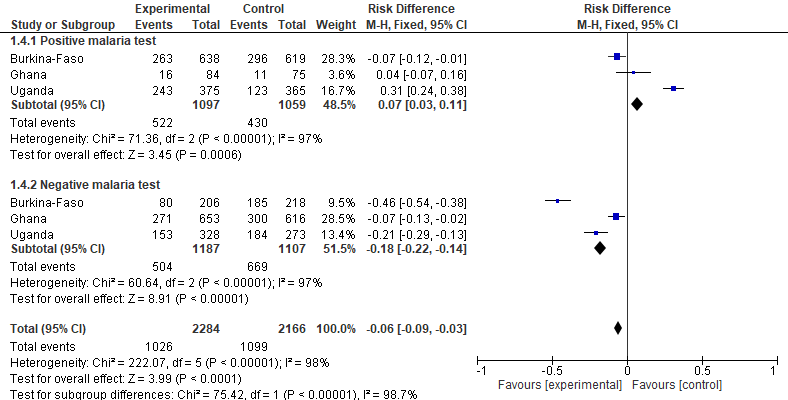


CI, confidence interval; M-H, Mantel-Haenszel method.

SF5. Antibiotic prescriptions by respiratory and malaria status – relative (A) and absolute (B) effects

A


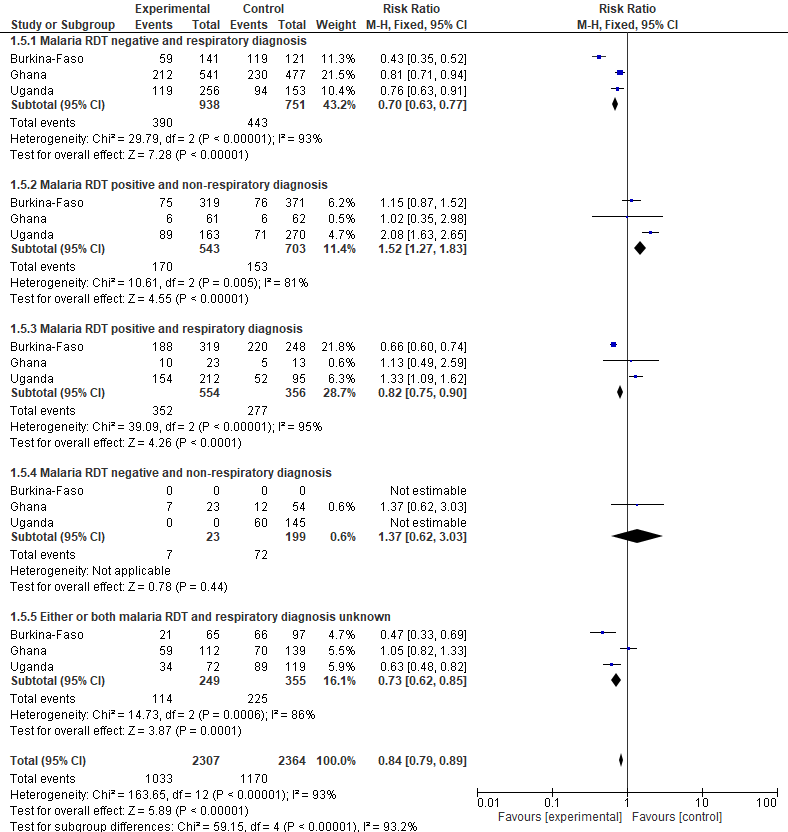


CI, confidence interval; M-H, Mantel-Haenszel method; RDT, rapid diagnostic test.

B


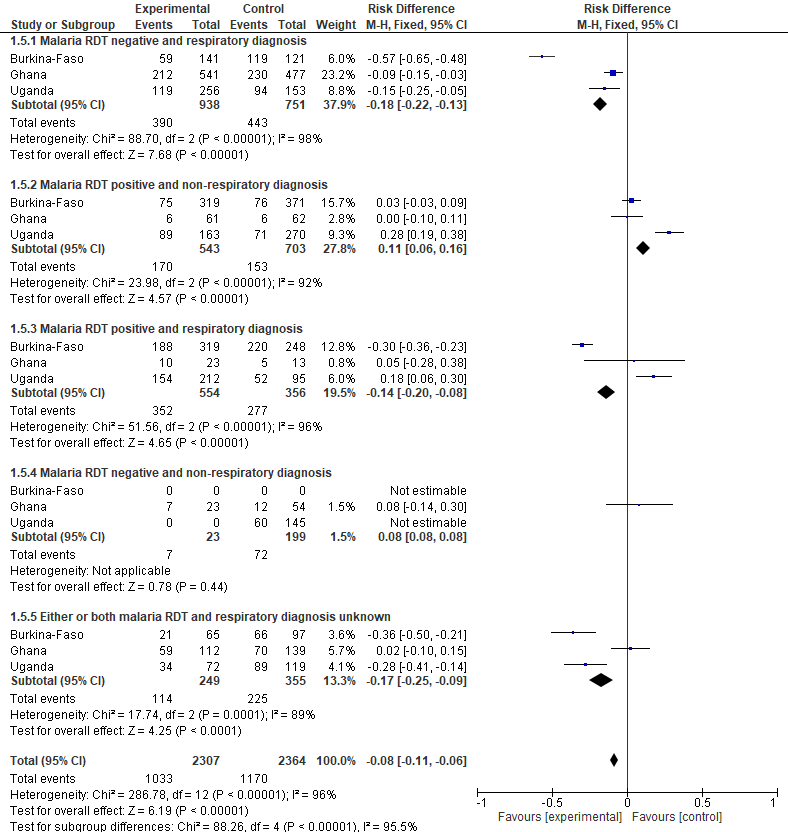


CI, confidence interval; M-H, Mantel-Haenszel method; RDT, rapid diagnostic test.

SF6. Antibiotic prescriptions by respiratory and malaria status in under 5’s – relative (A) and absolute (B) effects

A


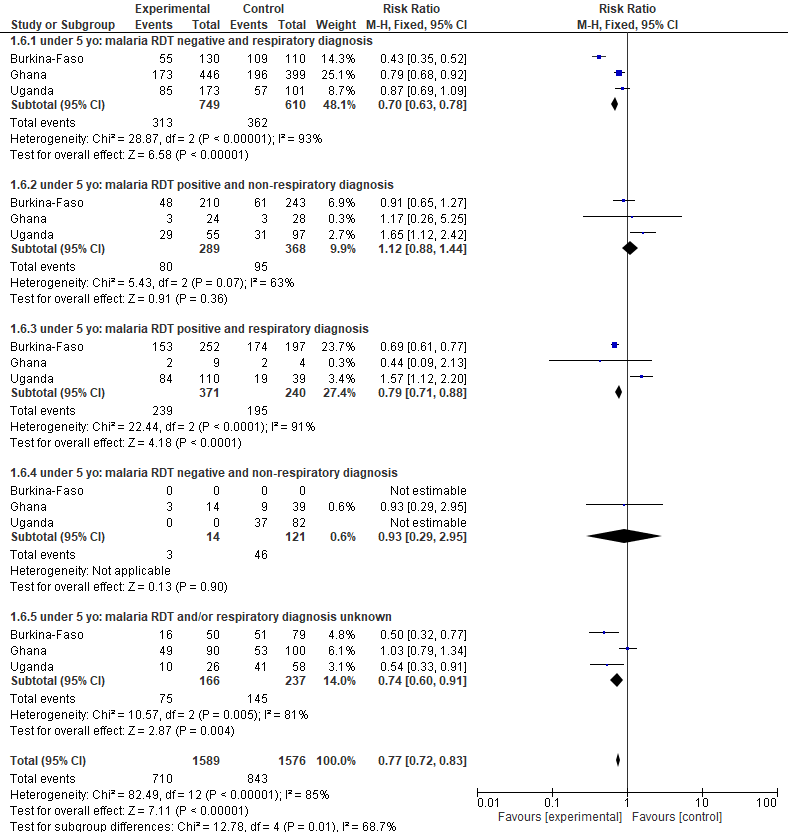


CI, confidence interval; M-H, Mantel-Haenszel method; RDT, rapid diagnostic test; yo, years old.

B


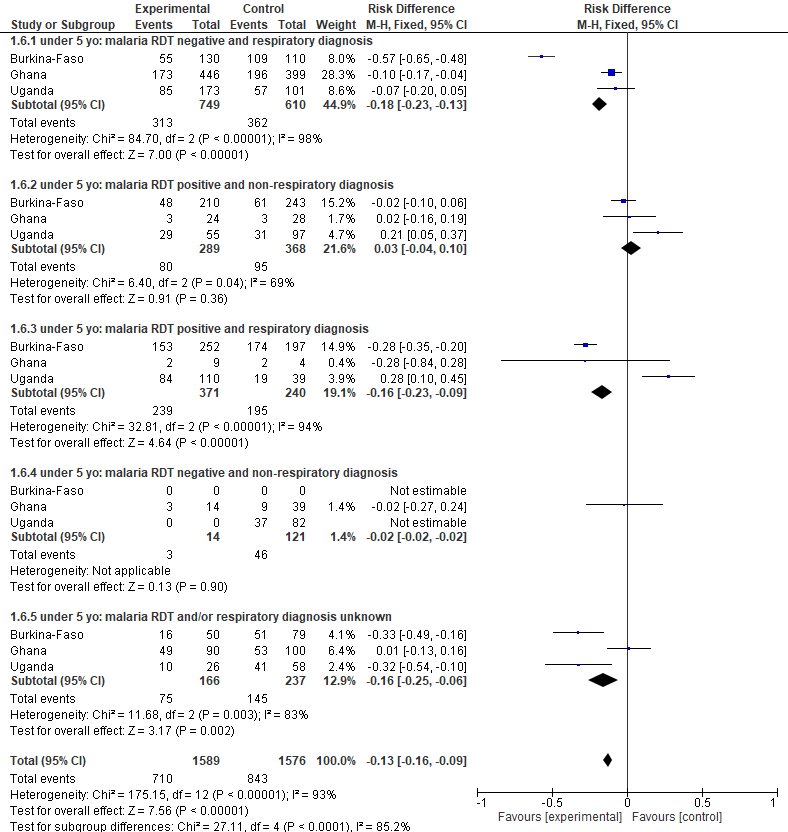


CI, confidence interval; M-H, Mantel-Haenszel method; RDT, rapid diagnostic test; yo, years old.

SF7. C-reactive protein levels (mg/L) in patients enrolled in the intervention arm with malaria RDT-positive (left) and RDT-negative (right) test


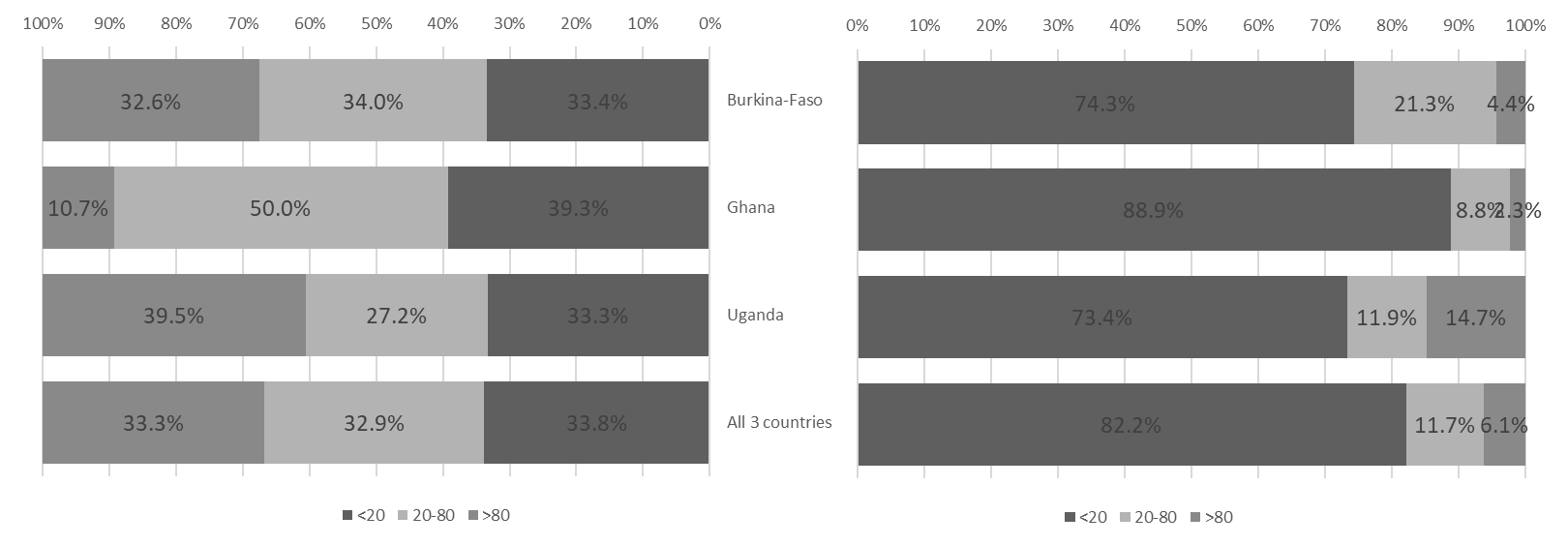


RDT, rapid diagnostic test.

## Supplementary tables

ST1. Seasonal variability of malaria tests

Q, quarter.

ST2. Antibiotic prescriptions by yearly quarter – relative and absolute effects

Q, quarter.

ST3. Use and results of rapid diagnostic tests (RDTs) in the intervention arm

|  | Burkina Faso | | Ghana | | Uganda | | All three African countries | |
| --- | --- | --- | --- | --- | --- | --- | --- | --- |
| Test | done n(%) | positive n(%) | done n(%) | positive n(%) | done n(%) | positive n(%) | done n(%) | positive n(%) |
| Malaria | 856 (100%) | 646 (75.5%) | 738 (97.0%) | 84 (11.4%) | 703 (100%) | 375 (53.3%) | 2297 (99.0%) | 1105 (48.1%) |
| Typhoid | 279 (32.6%) | 8 (2.9%) | 759 (99.7%) | 12 (1.6%) | 381 (54.2%) | 36 (9.4%) | 1419 (61.2%) | 56 (3.9%) |
| GAS | 36 (4.2%) | 2 (5.6%) | 758 (99.6%) | 35 (4.6%) | 167 (23.8%) | 32 (19.2%) | 961 (41.4%) | 69 (7.2%) |
| Flu | 379 (44.3%) | 23 (6.1%) | 758 (99.6%) | 76 (10.0%) | 477 (67.9%) | 26 (5.5%) | 1614 (69.6%) | 125 (7.7%) |
| RSV (<5yrs) | 136 (15.9%) | 3 (2.2%) | 755 (99.2%) | 21 (2.8%) | 140 (19.9%) | 8 (5.7%) | 1031 (44.4%) | 32 (3.1%) |
| *S. pneumoniae* Urine (>5yrs) | 24 (2.8%) | 3 (12.5%) | 412 (54.1%) | 21 (5.1%) | 324 (46.1%) | 45 (13.9%) | 760 (32.8%) | 69 (9.1%) |
| WBC Esterase Urine | 1 (0.1%) | 0 (0.0%) | 399 (52.4%) | 57 (14.3%) | 90 (12.8%) | 38 (42.2%) | 490 (21.1%) | 95 (19.4%) |
| WBC Nitrites Urine | 5 (0.6%) | 0 (0.0%) | 401 (52.7%) | 29 (7.2%) | 91 (12.9%) | 3 (3.3%) | 497 (21.4%) | 32 (6.4%) |
|  | done n(%) | median (Q1,Q3) | done n(%) | median (Q1,Q3) | done n(%) | median (Q1,Q3) | done n(%) | median (Q1,Q3) |
| CRP mg/L | 788 (92%) | 28.6 (6, 84) | 751 (98.7%) | 1.7 (1, 11.8) | 702 (99.9%) | 17.3 (1, 95) | 2241 (96.6%) | 11.5 (1, 49.4) |
| WBC counts (x 1000) | 787 (92%) | 9.3 (7.2, 11.9) | 761 (100%) | 8.7 (6.6, 11.6) | 701 (99.7%) | 8.1 (6.1, 10.2) | 2249 (96.9%) | 8.7 (6.7, 11.3) |
| Neutrophil counts (%) | 788 (92%) | 55 (41, 66) | 762 (100%) | 45 (34, 58) | 700 (99.6%) | 39 (29, 50) | 2248 (96.9%) | 46 (34, 59) |

CRP, C-reactive protein; GAS, group A streptococci; RSV, respiratory syncytial virus; WBC, white blood cell count.

ST4. Antibiotic prescriptions in the presence of a positive test result for the detection of bacteria or viruses

| RDT | Country | Positive RDT | No antibiotic | Antibiotic prescribed | % Antibiotic |
| --- | --- | --- | --- | --- | --- |
| typhoid | Burkina Faso | 8 | 0 | 8 | 100.0% |
|  | Ghana | 12 | 2 | 10 | 83.3% |
|  | Uganda | 36 | 0 | 36 | 100.0% |
|  | All 3 countries | 56 | 2 | 54 | 96.4% |
| GAS | Burkina Faso | 2 | 0 | 2 | 100.0% |
|  | Ghana | 34 | 8 | 26 | 76.5% |
|  | Uganda | 32 | 1 | 31 | 96.9% |
|  | All 3 countries | 68 | 9 | 59 | 86.8% |
| S.pneumoniae | Burkina Faso | 3 | 0 | 3 | 100.0% |
|  | Ghana | 21 | 3 | 18 | 85.7% |
|  | Uganda | 45 | 0 | 45 | 100.0% |
|  | All 3 countries | 69 | 3 | 66 | 95.7% |
| WBC esterase | Burkina Faso | 0 | 0 | 0 |  |
|  | Ghana | 54 | 29 | 25 | 46.3% |
|  | Uganda | 38 | 2 | 36 | 94.7% |
|  | All 3 countries | 92 | 31 | 61 | 66.3% |
| WBC nitrates | Burkina Faso | 0 | 0 | 0 |  |
|  | Ghana | 29 | 12 | 17 | 58.6% |
|  | Uganda | 3 | 1 | 2 | 66.7% |
|  | All 3 countries | 32 | 13 | 19 | 59.4% |
| Flu | Burkina Faso | 23 | 17 | 6 | 26.1% |
|  | Ghana | 70 | 35 | 35 | 50.0% |
|  | Uganda | 26 | 14 | 12 | 46.2% |
|  | All 3 countries | 119 | 66 | 53 | 44.9% |
| RSV | Burkina Faso | 3 | 0 | 3 | 100.0% |
|  | Ghana | 21 | 13 | 8 | 38.1% |
|  | Uganda | 8 | 2 | 6 | 75.0% |
|  | All 3 countries | 32 | 15 | 17 | 53.1% |

CRP, C-reactive protein; GAS, group A streptococci; RDT, rapid diagnostic test; RSV, respiratory syncytial virus; WBC, white blood cell count.

ST5. Clinical outcomes and antibiotic prescriptions stratified by CRP, WBC total counts and neutrophil counts.

|  |  | Test result |  | Favourable clinical outcome | | Antibiotic prescriptions | |
| --- | --- | --- | --- | --- | --- | --- | --- |
|  |  | n/N (%) | 95% CI | n/N (%) | 95% CI | n/N (%) | 95% CI |
| C-reactive protein (CRP) | Burkina Faso | 777/856 (90.8) |  |  |  |  |  |
|  | <20 | 333 (42.9) |  | 332/333 (99.7) | 98.3–100.0 | 87/333 (26.1) | 21.7–31.1 |
|  | 20 to <80 | 243 (31.3) |  | 242/243 (99.6) | 97.7–99.9 | 73/243 (30.0) | 24.6–36.1 |
|  | >=80 | 201 (25.9) |  | 199/201 (99.0) | 96.5–99.7 | 117/201 (58.2) | 51.3–64.8 |
|  | Ghana | 750/761 (98.6) |  |  |  |  |  |
|  | <20 | 627 (83.6) |  | 625/627 (99.7) | 98.8–99.9 | 228/627 (36.4) | 32.7–40.2 |
|  | 20 to <80 | 99 (13.2) |  | 99/99 (100.0) | 96.3–100.0 | 43/99 (43.4) | 34.1–53.3 |
|  | >=80 | 24 (3.2) |  | 24/24 (100.0) | 86.2–100.0 | 17/24 (70.8) | 50.8–85.1 |
|  | Uganda | 702/703 (99.9) |  |  |  |  |  |
|  | <20 | 365 (52.0) |  | 336/365 (92.1) | 88.8-94.4 | 131/365 (35.9) | 31.1–40.9 |
|  | 20 to <80 | 143 (20.4) |  | 140/143 (97.9) | 94.0–99.3 | 74/143 (51.7) | 43.6–59.8 |
|  | >=80 | 194 (27.6) |  | 185/194 (95.4) | 91.4–99.3 | 191/194 (98.5) | 95.5–99.5 |
|  | All 3 countries | 2229/2320 (96.1) |  |  |  |  |  |
|  | <20 | 1325 (59.4) |  | 1293/1325 (97.6) | 96.6–98.3 | 446/1325 (33.7) | 31.2–36.2 |
|  | 20 to <80 | 485 (21.8) |  | 481/485 (99.2) | 97.9–99.7 | 190/485 (39.2) | 34.9–43.6 |
|  | >=80 | 419 (18.8) |  | 408/419 (97.4) | 95.4–98.5 | 325/419 (77.6) | 73.3–81.3 |
| White blood cell counts | Burkina Faso | 776/856 (90.7) |  |  |  |  |  |
|  | <11,000 | 524 (67.5) |  | 522/524 (99.6) | 98.6–99.9 | 136/524 (26.0) | 22.4–29.9 |
|  | >=11,000 | 252 (32.5) |  | 250/252 (99.2) | 97.2–99.8 | 141/252 (56.0) | 49.8–62.0 |
|  | Ghana | 760/761 (99.9) |  |  |  |  |  |
|  | <11,000 | 540 (71.1) |  | 538/540 (99.6) | 98.7–99.9 | 166/540 (30.7) | 27.0–34.8 |
|  | >=11,000 | 220 (28.9) |  | 220/220 (100.0) | 98.3–100.0 | 128/220 (58.2) | 51.6–64.5 |
|  | Uganda | 701/703 (99.7) |  |  |  |  |  |
|  | <11,000 | 562 (80.2) |  | 530/562 (94.3) | 92.1–95.9 | 303/562 (53.9) | 49.8–58.0 |
|  | >=11,000 | 139 (19.8) |  | 130/139 (93.5) | 88.1–96.6 | 92/139 (66.2) | 58.0–73.5 |
|  | All 3 countries | 2237/2320 (96.4) |  |  |  |  |  |
|  | <11,000 | 1626 (72.7) |  | 1590/1626 (97.8) | 97.0–98.4 | 605/1626 (37.2) | 34.9–39.6 |
|  | >=11,000 | 611 (27.3) |  | 600/611 (98.2) | 96.8–99.0 | 361/611 (59.1) | 55.1–62.9 |
| Neutrophils counts | Burkina Faso | 776/856 (90.7) |  |  |  |  |  |
|  | <75% | 706 (91) |  | 702/706 (99.4) | 98.6–99.8 | 232/706 (32.9) | 29.5–36.4 |
|  | >=75% | 70 (9) |  | 70/70 (100.0) | 94.8–100.0 | 45/70 (64.3) | 52.6–74.5 |
|  | Ghana | 760/761 (99.9) |  |  |  |  |  |
|  | <75% | 714 (93.9) |  | 712/714 (99.7) | 99.0–99.9 | 268/714 (37.5) | 34.1–41.1 |
|  | >=75% | 46 (6.1) |  | 46/46 (100.0) | 92.3–100.0 | 26/46 (56.5) | 42.2–69.8 |
|  | Uganda | 700/703 (99.6) |  |  |  |  |  |
|  | <75% | 695 (99.3) |  | 654/695 (94.1) | 92.1–95.6 | 389/695 (56.0) | 52.3–59.6 |
|  | >=75% | 5 (0.7%) |  | 5/5 (100.0) | 56.5–100.0 | 5/5 (100.0) | 56.5–100.0 |
|  | All 3 countries | 2236/2320 (96.4) |  |  |  |  |  |
|  | <75% | 2115 (94.6) |  | 2068/2115 (97.8) | 97.1–98.3 | 889/2115 (42.0) | 40.0–44.1 |
|  | >=75% | 121 (5.4) |  | 121/121 (100.0) | 96.9–100.0 | 76/121 (62.8) | 53.9–70.9 |

CI, confidence interval; CRP: C-reactive protein; RDT, rapid diagnostic test; RSV: respiratory syncytial virus; WBC: white blood cell count.
